# Extracellular Vesicles as Next-Generation Biomarkers in Lung Cancer Patients: A Case Report on Adenocarcinoma and Squamous Cell Carcinoma

**DOI:** 10.3390/life14030408

**Published:** 2024-03-20

**Authors:** Monika Ruzycka-Ayoush, Monika Prochorec-Sobieszek, Andrzej Cieszanowski, Maciej Glogowski, Anna Szumera-Cieckiewicz, Joanna Podgorska, Alicja Targonska, Kamil Sobczak, Grazyna Mosieniak, Ireneusz P. Grudzinski

**Affiliations:** 1 Faculty of Pharmacy, Medical University of Warsaw, Banacha 1, 02-097 Warsaw, Poland; ireneusz.grudzinski@wum.edu.pl; 2 Department of Cancer Pathomorphology, Maria Sklodowska-Curie National Research Institute of Oncology, Roentgena Str. 5, 02-781 Warsaw, Poland; monika.prochorec-sobieszek@wum.edu.pl (M.P.-S.); anna.szumera-cieckiewicz@pib-nio.pl (A.S.-C.); 3 Department of Clinical Radiology, Maria Sklodowska-Curie National Research Institute of Oncology, Roentgena Str. 5, 02-781 Warsaw, Poland; andrzej.cieszanowski@wum.edu.pl (A.C.); joanna.podgorska@wum.edu.pl (J.P.); 4 Department of Lung Cancer and Chest Tumors, Maria Sklodowska-Curie National Research Institute of Oncology, Roentgena Str. 5, 02-781 Warsaw, Poland; maciej.glogowski@pib-nio.pl; 5 Laboratory of Molecular Bases of Ageing, Nencki Institute of Experimental Biology, Polish Academy of Sciences, 02-093 Warsaw, Poland; a.targonska@nencki.edu.pl (A.T.); g.mosieniak@nencki.edu.pl (G.M.); 6 Faculty of Chemistry, Biological and Chemical Research Centre, University of Warsaw, Zwirki i Wigury 101, 02-089 Warsaw, Poland; ksobczak@cnbc.uw.edu.pl

**Keywords:** lung cancer, extracellular vesicles, surface proteins, biomarkers, diagnosis

## Abstract

Extracellular vesicles (EVs) released from primary cell lines, originating from resected tissues during biopsies in patients with non-small cell lung cancer (NSCLC) revealing adenocarcinoma and squamous cell carcinoma subtypes, were examined for membrane proteomic fingerprints using a proximity barcoding assay. All the collected EVs expressed canonical tetraspanins (CD9, CD63, and CD81) highly coexpressed with molecules such as lysosome-associated membrane protein-1 (LAMP1–CD107a), sialomucin core protein 24 (CD164), Raph blood group (CD151), and integrins (ITGB1 and ITGA2). This representation of the protein molecules on the EV surface may provide valuable information on NSCLC subtypes and offer new diagnostic opportunities as next-generation biomarkers in personalized oncology.

## 1. Introduction

Extracellular vesicles (EVs) are part of the cell “secretome” participating in intercellular communication. They facilitate the transfer of biological signals such as non-coding RNAs, coding RNAs, DNA strands, portions, and lipids to recipient cells [1,2,3]. Numerous biological effects of cancer cells rely on their “secretome” and more specifically nanosized EVs (30–150 nm), including exosomes [4,5,6]. Because they can recapitulate a substantial part of the parent cancer cell’s biological effects, playing a pivotal role in a range of cellular processes such as proliferation, growth, development, angiogenesis, metastasis, reprogramming, and remodeling, these endogenous nanoparticles are considered potential diagnostics and therapeutic targets in modern nanomedicine [4,5,6].

Surface proteins located on extracellular vesicles are understood to have significant biological roles for regulating EV homing, targeting, and uptake by recipient cells [6]. Note that most surface proteins are adhesive molecules [7,8] that facilitate effective EV communication by binding to the target receptors on the recipient cell membranes [2]. The most commonly recognized EVs surface proteins are tetraspanins such as CD9, CD63, and CD81, which are also recognized as canonical biomarkers [9]. Additionally, cytokines, phosphatidylserine, proteoglycans, and numerous adhesive molecules such as integrins have been also identified on the EV surface [10]. Since the membrane of EVs generally mirrors the membrane of the cancer cell from which the EVs are originated, the altered profile of specific surface proteins on tumor-released EVs may provide novel insights into the cancer and its environments.

While there is considerable interest in utilizing various RNA species (such as mRNA, miRNA, lncRNA, and other RNA), DNA (mtDNA, ssDNA, and dsDNA), and certain lipids as EV biomarkers in non-small cell lung cancer (NSCLC), there are limited data supporting the use of EV surface proteins as novel biomarkers in different NSCLC subtypes.

Recently, a high-throughput proximity barcoding assay (PBA) to simultaneously profile over hundreds of surface proteins for their presence on individual exosomes has been developed [1]. This method employs a unique DNA motif, created via rolling circle amplification (RCA), for the barcoding of individual exosomes. The protein composition on a single exosome’s surface is transformed into DNA sequence data by bound antibody–DNA conjugates, which include a random tag sequence repeated in each RCA product. Following PCR amplification, the protein and exosome identity details encoded in the DNA strand are deciphered using next-generation sequencing to distinguish the surface protein composition of individual exosomes [1].

Given that the composition of EV-borne surface proteins (EVSPs) can reflect the state of the cancer cell [7,11], we established primary cancer cell lines from resected lung cancer tissues obtained through biopsies from lung cancer patients. These cell lines were used to produce cancer-born EVs, which were characterized based on EVSP using the PBA technology in two NSCLC subtypes, namely lung adenocarcinoma (LA) and squamous cell carcinoma (SCC). This representation of the proteomic landscape on EVs may provide valuable information and present new diagnostic opportunities as next-generation proteomic biomarkers in personalized oncology.

## 2. Materials and Methods

### 2.1. Patients

**Figure 1:**
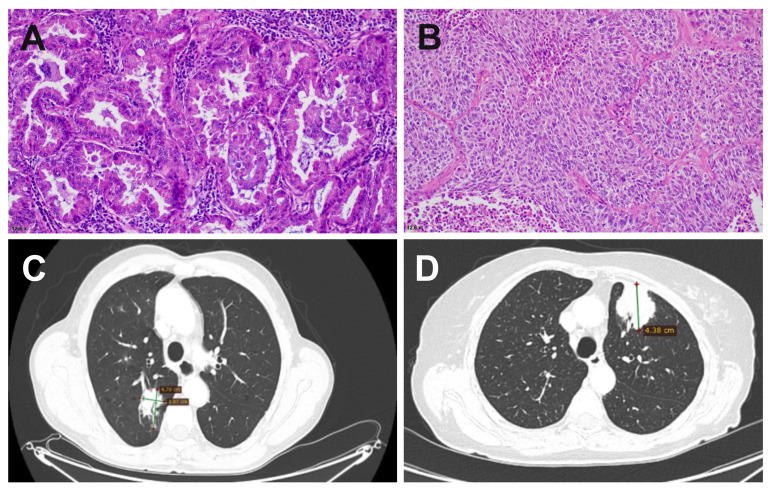
Histopathology specimens (**A**,**B**) and computed tomography (CT) images (**C**,**D**) of non-small cell lung cancer (NSCLC) in patients H22/987 and H22/518 revealing adenocarcinoma (**A**,**C**) and squamous cell carcinoma (**B**,**D**). The histology stain with H&E (mag. ×200). The CT images were performed on a 64-detector CT scanner (Revolution EVO, GE medical systems, Chicago, IL, USA) with the use of helical mode, slice thickness 2.5 mm, spacing 2.5 mm, spiral pitch factor of 0.984375, single collimation width 0.625, total collimation width 40, X-ray tube current 130 mA, tube voltage 120 kV. For contrast enhancement, Ultravist 370 (Bayer Pharma, Berlin, Germany) was administered intravenously using a power injector in a dose of 80 mL.

The Bioethics Committee of the Maria Sklodowska-Curie National Research Institute of Oncology granted ethical approval for this study (reference: 35/2020). The study involved five patients, comprising four females and one male, with ages ranging between 62 and 78. All patients underwent contrast-enhanced computed tomography (CT) of the chest for preoperative tumor staging. Representative CT images are shown in Figure 1. Tumor staging was determined according to the 8th edition of the pTNM classification (Table 1) [12].

### 2.2. Clinical Examination

To establish the diagnosis, resection specimens, including lobectomy and segmentectomy, underwent histopathological examination. Tissues were fixed in 10% formalin, processed routinely, and stained with hematoxylin and eosin (Figure 1). The diagnosis was performed by a certified pathologist following the 2021 WHO classification of thoracic tumors (Table 1) [13].

Qualification for the collection of lung cancer tissues was conducted by a clinician through a multifactor assessment of the patient’s condition. Inclusion criteria comprised patients with a cytologically or histopathologically confirmed diagnosis of NSCLC and a tumor size greater than 1 cm, as determined by CT images.

### 2.3. Sampling

A 1 cm × 1 cm × 1 cm cube fragment was excised from the surgically removed tumor tissue under sterile conditions and immediately placed in a transport medium. The sample was transported on ice to the cell culture laboratory. Subsequently, a tiny fragment of the collected cancer tissues was used to set up the primary cancer cell lines for the production of EVs.

### 2.4. EVs Isolation

The cell culture media obtained from the primary cell lines was first centrifuged at 750× *g* at 4 °C for 15 min to eliminate the detached cells. Subsequently, the collected media underwent centrifugation at 2000× *g* at 4 °C for 20 min to remove the microvesicles. The supernatant was then filtered through 0.45 µm filters and subsequently spun in a Beckman Coulter Optima™ L-80XP Ultracentrifuge (Beckman Coulter, Brea, CA, USA) at 10,000× *g* at 4 °C for 45 min using a Type SW 32 Ti rotor (Beckman Coulter, Brea, CA, USA) to remove the apoptotic bodies and cell debris. Afterwards, the supernatant was once again collected, filtered through 0.22 µm filters and ultracentrifuged at 100,000× *g* at 4 °C for 90 min to pellet the EVs. Subsequently, the supernatant was eliminated, and the raw pellets containing EVs were resuspended in a portion of PBS and combined [14].

### 2.5. EVs Characterization

Transmission Electron Microscopy (TEM). Purified EV preparations underwent fixation in a 4% aqueous solution of paraformaldehyde, which was followed by rinsing in deionized water. Subsequently, they were placed onto a carbon precoated 300-mesh copper grid for 1 h, and staining was performed using the UA-Zero® EM Stain (Agar Scientific Ltd., Stansted, Essex, UK). The morphology of the isolated EVs was examined via TEM utilizing a transmission electron microscope TALOS F200X (FEI, currently Thermo Fisher Scientific). 

Nanoparticle Tracking Analysis (NTA). The mean size of EVs were assessed utilizing a NanoSight NS300 (Malvern Panalytical Ltd., Malvern, UK) fitted with a 488 nm blue laser. Five 30 s videos were recorded for each measurement and analyzed employing the integrated NanoSight Software NTA 3.2. Before measurement, each sample was diluted in PBS at a ratio of 1:4.

Quantification of EVSP (BCA). The protein content of EVs derived from primary cell lines was determined using the BCA method (Pierce™ BCA Protein Assay kit, Thermo Fisher Scientific, Waltham, MA, USA), following the manufacturer’s protocol. Prior to protein quantification, the EVs suspended in cryoprotectant were lysed by combining them with an equal volume of a mixture containing RIPA buffer (Pierce™ RIPA Buffer, Thermo Fisher Scientific, USA) and Halt™ Protease Inhibitor Cocktail (Thermo Fisher Scientific, USA). Sonication was performed for 10 min on ice to facilitate lysis. The absorbance was then measured at 562 nm using an Epoch microplate reader (BioTek Inc., New Castle, DE, USA).

Profiling EVSP with a high-throughput proximity barcoding assay (PBA). The characterization of individual EVSP involved conducting PBA with antibody–DNA conjugates and subsequent next-generation sequencing in accordance with the details provided in the cited literature [1,15]. The preparation of PBA probes consisted of attaching the antibody to DNA oligonucleotides and a unique molecular identifier (UMI). The DNA oligonucleotides contained a protein tag that identifies the specific surface protein of EVs. UMI is a molecular tag responsible for distinguishing individual protein molecules after PCR amplification and DNA sequencing. Then, to barcode individual EVs, complex tags embedded in circular DNA molecules were subjected to rolling circle amplification (RCA). These complex tags were integrated into the antibody-conjugated oligonucleotides, serving as markers for proteins on single EVs that have colocalized with unique RCA products [1,15].

### 2.6. Statistical Analysis

Statistical analysis was performed using either Statistica 13.3 or GraphPad Prism 9.3.0 (GraphPad Prism Software), employing tests as specified in the figure legends. Normal distribution was assessed using Shapiro–Wilk tests. To evaluate the statistical significance between two independent variables, a two-way ANOVA was conducted, which was followed by Tukey’s multiple comparisons test. *p*-values less than 0.05 were considered representing statistically significant differences. The log_2_-transformed circular heatmap with a dendrogram with squared Euclidean distance was performed using OriginPro, Version 2022b (OriginLab Corporation, Northampton, MA, USA).

## 3. Results and Discussion

**Figure 2:**
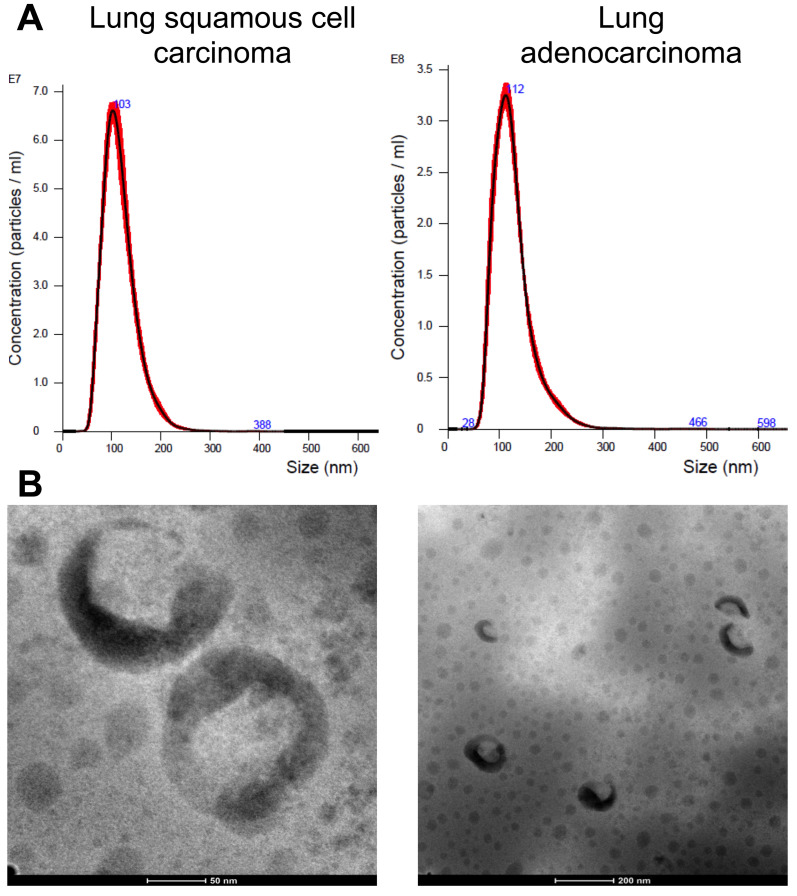
(**A**) The representative plot of the size and concentration of lung cancers-derived EVs suspended in PBS. (**B**) HAADF image of the EVs from lung adenocarcinoma (LA) and lung squamous cell carcinoma (SCC).

The confirmation of EVs presence was achieved through TEM and NTA, as illustrated in Figure 2. The observed EVs exhibited an average diameter of 120 ± 3 nm. Moreover, total protein concentration determination revealed an average concentration of 645 ± 66 µg mL^−1^ across all samples.

**Figure 3:**
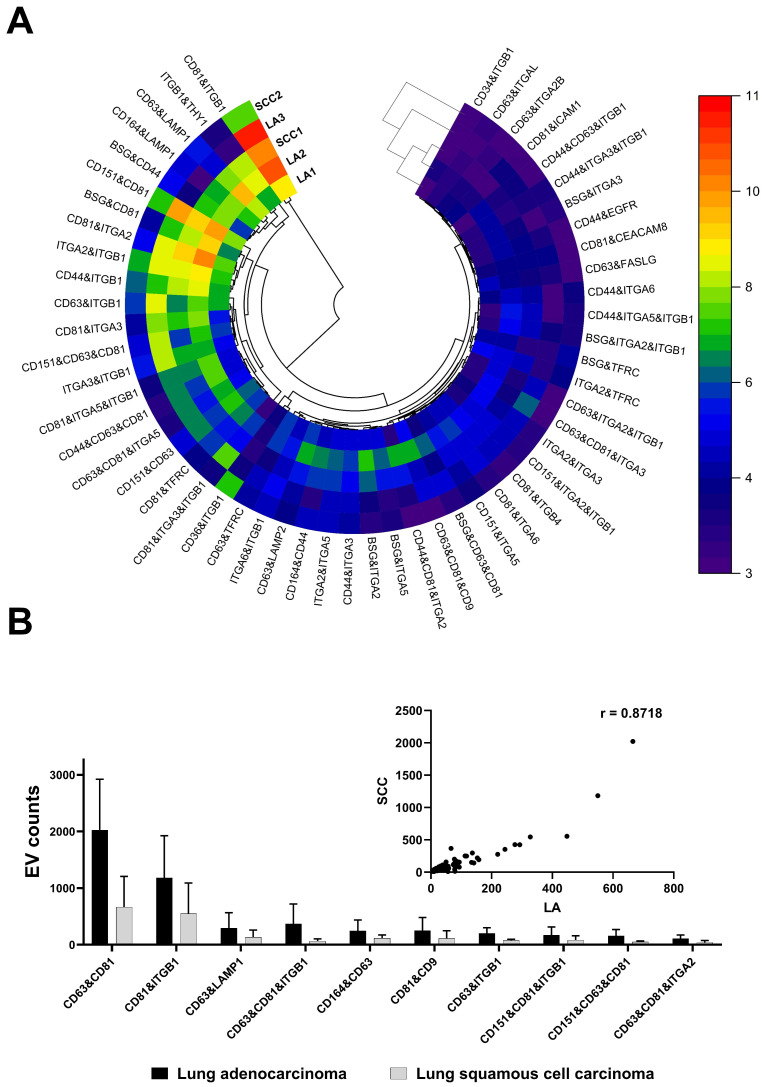
Proteomic fingerprints of extracellular vesicles derived from primary cell lines established based on NSCLC cells representing lung adenocarcinoma (LA) and lung squamous cell carcinoma (SCC) in patients. The EVSP profile was shown as the log_2_-transformed circular heatmap with dendrogram with squared Euclidean distance (**A**). The major EVSPs counts and the scatter plot of Spearman correlation (insert) for the major protein clusters expressed in the LA and SCC (**B**).

Subsequent analysis involved the examination of surface proteins on individual EVs released from primary cell lines originating from resected tissues during biopsies in patients with NSCLC, which were identified as both LA and SCC subtypes. Employing proximity barcoding technology, we detected an average count of 173,250 ± 31,694 EVs. This thorough investigation uncovered the presence of at least 181 surface proteins representing various groups of transmembrane receptors, as depicted in Figure 3A.

Among the identified surface proteins released from NSCLC cells, canonical exosomal tetraspanins emerged as predominant, notably the CD63 receptor—a cell surface binding partner for TIMP-1—and the CD81 proteins, known as a target of the antiproliferative antibody 1 (TAPA-1), also referred to as tetraspanin-28 (Tspan-28). Notably, CD81 expression was particularly pronounced in lung adenocarcinoma (Figure 3A). These markers, alongside CD9 (Tspan29), CD37 (Tsapn26), and CD82 (Tspan 27) proteins, belong to the tetraspanin family, which is recognized as the transmembrane 4 superfamily. Most members of this family are cell-surface proteins characterized by four hydrophobic transmembrane domains that can anchor multiple proteins to a region of the cell membrane, forming four-transmembrane protein-enriched microdomains [16]. Tetraspanins mediate signal transduction events involved in the regulation of immune responses, cell development, activation, growth, motility, and fusion. Recent studies have evidence that the downregulation of CD9, CD63, or CD81 protein levels correlates with tumor progression and metastasis in solid tumors [16]. Notably, among the tetraspanins, the CD9 protein expression was selectively downregulated in small cell lung cancer (SCLC) but not in NSCLC, which was potentially linked to the more aggressive phenotype of SCLC.

In addition to the canonical EV-specific tetraspanins, several other surface proteins were identified in clusters, including CD151, CD164, ITGB1, ITGA2, and LAMP1 proteins (Figure 3A). Interestingly, the CD151 molecule (Raph blood group) is known to be expressed in various cancer cells and endothelial cells, exerting regulatory effects on postadhesion events such as cell spreading, migration, invasion, metastasis, and tumor neovascularization [17]. The molecular mechanism of CD151 in cancer involves its ability to regulate the function of interacting proteins [17]. CD151 directly interacts with integrins, such as α3β1 and α6β4, influences the activity of growth factors (EGFR, TGF-β), and regulates the expression and activity of matrix metalloproteinases, especially MMP2 and MMP9 [17,18]. Overexpression of the CD151 molecule has been associated with poor prognosis in lung cancers.

Another significant group of surface proteins detected on EVs derived from primary lung cancer cells consists of integrin subunits, including ITGB1 (integrin β1) and ITGA2 (integrin α2). Integrins, a family of cell surface receptors, play a crucial role in cell adhesion and signaling, facilitating cell-to-cell and cell-to-extracellular matrix (ECM) communication [19,20,21]. Comprising alpha and beta subunits, each responsible for different biological functions [21], integrins present on tumor cells or tumor-associated stromal cells have been involved in cancer metastasis, stemness, and drug resistance [20]. The subunit β1-containing integrins represent the largest subgroup and are found to be overexpressed in lung, breast, and colorectal cancers [22].

Interestingly, it has been suggested that the function of β1 integrin is modified by the CD9 molecule in cancer cells, resulting in reduced motility. In this study, an increase in ITGB1 expression was observed on EVs released from lung adenocarcinoma compared to squamous cell carcinoma (Figure 3B). It is noteworthy that ITGB1 undergoes alterations during tumorigenesis [23], mediating tumor resistance to various anticancer drugs and inducing radiation resistance in NSCLC [24]. Additionally, a slightly elevated expression of ITGA2 was noted in the cluster of the LA compared to the SCC (Figure 3B). The overexpression of ITGA2 in lung cancer cells is associated with increased adhesion and migration capacity [23], while clinical samples show a correlation with shorter relapse-free survival [23]. As for anticancer therapy, proteins encoded by ITGB1 and ITGA2 genes are considered as cancer-related genes, with ITGB1 being an FDA-approved drug target [25].

Significant amounts of membrane proteins, including lysosome-associated membrane protein-1 (LAMP1) and sialomucin core protein 24 (CD164), were also detected on the surface of EVs derived from primary NSCLC cells (Figure 3A). The dysregulation of LAMP1 has been observed in cancers and is linked to tumor growth, promotion, and metastasis [26]. Moreover, emerging evidence suggests that increased CD164 expression is correlated with aggressive metastasis, advanced stages, and shorter overall survival in lung cancer. The recognition of CD164 as a potential cancer stem cell therapeutic marker holds promise for developing effective therapy in patients with chemoresistant lung cancer [27].

Utilizing proximity barcoding technology, we identified a diverse array of surface proteins on individual EVs, including canonical tetraspanins (CD9, CD63, and CD81), integrins (ITGB1 and ITGA2), and other molecules such as CD151, CD164, and LAMP1. These proteins constitute a major EVSP fingerprint observed in both LA and SCC subtypes, highlighting their potential as diagnostic targets in NSCLC. Furthermore, our findings suggest potential subtype-specific differences in protein expression patterns, with differences in the expression of ITGB1 and ITGA2 between LA and SCC, which may be related to tumor aggressiveness, metastasis, and response to therapy.

However, our study has some limitations, which are mainly due to the limited number of samples resulting from challenges in obtaining human lung cancer tissues. This limitation hinders the ability to distinguish trends in EV surface protein expression levels among different cancer subtypes comprehensively. Additionally, while our investigation primarily focused on tissue-derived EVs, we recognize the need for further research exploring the use of easier-to-collect materials such as whole blood, plasma, serum, or urine well known as liquid biopsies. Taking together the results from the as-performed analysis of EVs taken from surgically collected tissues with easy-to-collect materials could provide valuable insights into the clinical utility of EV-borne surface proteins as non-invasive biomarkers for NSCLC diagnosis, prognosis, and treatment response assessment.

## 4. Conclusions

We conducted an analysis of membrane proteins in extracellular vesicles released from primary cell lines established from non-small cell lung cancer in five patients. The obtained EVs expressed canonical tetraspanins (CD9, CD63, and CD81) along with molecules such as lysosome-associated membrane protein-1 (LAMP1–CD107a), sialomucin core protein 24 (CD164), Raph blood group (CD151), and integrins ITGB1 and ITGA2. This set of molecules constituted a major EVSPs fingerprint observed in both lung adenocarcinoma and lung squamous cell carcinoma representing potent patient-personalized EV-based biomarkers. However, further research in this area is required, particularly exploring the use of easier-to-collect materials such as liquid biopsies in lung cancer patients.

## Figures and Tables

**Table 1:** Lung cancer patients subjected to the study.

Patient	WHO Classification	Histopathologic Grade	Pathologic Stage Classification (pTNM)
H22/518 F/75	Squamous cell carcinoma	G3	pT3, N0, Mx, PL0, V0/L1, R0
H22/987 F/78	Adenocarcinoma	G1	pT2b, N0, Mx, PL0, L0/V0, R0
H22/3994 M/64	Squamous cell carcinoma	G2	pT2a, pN0, Mx PL1, L0/V0, R0
H22/9303 F/62	Adenocarcinoma	G2	pT2a, pN0, Mx PL1, L0/V0, R0
H22/3486 F/63	Adenocarcinoma	G2	pT1b, N0, Mx PL1, L0/V0, R0

## Data Availability

Data are contained within the article.
